# An unexpected, mild phenotype of glucocorticoid resistance associated with glucocorticoid receptor gene mutation case report and review of the literature

**DOI:** 10.1186/s12881-018-0552-6

**Published:** 2018-03-06

**Authors:** Ágnes Molnár, Attila Patócs, István Likó, Gábor Nyírő, Károly Rácz, Miklós Tóth, Beatrix Sármán

**Affiliations:** 1 2nd Department of Internal Medicine, Semmelweis University, Szentkirályi u. 46, Budapest, H-1088 Hungary; 2 Hungarian Academy of Sciences and Semmelweis University “Lendület” Hereditary Endocrine Tumours Research Group, Budapest, Hungary; 3 Department of Laboratory Medicine, Semmelweis University, Budapest, Hungary; 4 Hungarian Academy of Sciences and Semmelweis University Molecular Medicine Research Group, Semmelweis University – Hungarian Academy of Sciences, Budapest, Hungary

**Keywords:** Glucocorticoid resistance, Glucocorticoid receptor, Mutation

## Abstract

**Background:**

Glucocorticoid resistance is a rare, sporadic or familial condition caused by mutation of the gene encoding the glucocorticoid receptor (GR). Clinically it is characterized by symptoms developed due to local, tissue-specific, or generalized partial insensitivity to glucocorticoids.

**Case presentation:**

A 31-year-old woman was evaluated because of infertility at the Endocrine Unit of the 2nd Department of Medicine, Semmelweis University. During her laboratory investigations, elevated serum and salivary cortisol were observed which failed to be suppressed after administration of 1 mg dexamethasone. 24 h urinary cortisol was increased, but a normal midnight serum cortisol was detected suggesting a maintained circadian rhythm. Plasma dehydroepiandrosterone-sulfate and androstendione levels were also elevated. Repeated plasma ACTH measurements indicated slightly elevated or normal values. Bone mineral density was normal. All laboratory results confirmed the diagnosis of glucocorticoid resistance. Genetic counseling followed by Sanger sequencing of the coding region of the gene encoding human glucocorticoid receptor was performed and a missense mutation (Arg714Gln, R714Q) in a heterozygous form was detected. Following family screening, the same mutation was found in her clinically-healthy 35-year-old sister who had no fertility problems.This variant was not detected in more than 60 patients and controls tested either for glucocorticoid resistance or Cushing’s syndrome in our Laboratory and it was absent in Exome Variant Server, HumanGene Mutation Database and ExAC databases.

**Conclusions:**

Our case fulfils the diagnostic criteria of glucocorticoid resistance, also named Chrousos syndrome. The glucocorticoid receptor gene mutation detected in our patient has been already reported in a 2-year-old child with hypoglycaemia, hypokalaemia, hypertension and premature puberty. These distinct phenotypes may suggest that other factors may modify the functional consequences of the R714Q variant of *GR.*

## Background

Glucocorticoids are essential steroid hormones involved in the regulation of adaptation to stress, carbohydrate-, protein-, fat-, calcium- and bone-metabolism, immune function, growth and behavioural regulation. They exert their actions through the glucocorticoid receptor (GR). The glucocorticoid receptor gene (*GR, NR3C1*) is located on chromosome 5q31 and contains 9 exons. The protein coding part starts from the second exon. The receptor consists of distinct domains including the N-terminal domain (NTD), the central DNA binding domain and the C-terminal ligand binding domain (LBD) [1, 2]. In the absent of ligand, GR is located in the cytoplasm in a multi-protein complex containing heat shock proteins and other chaperons [3]. Upon ligand binding, GR is released from this complex and translocates into the nucleus. In the nucleus it binds to the DNA through specific DNA sequences (GRE - glucocorticoid response element) and regulates the transcription of target genes [2, 4, 5]. The LBD domain contains a ligand-dependent activation function (AF-2), whose conformational change upon agonist binding stabilizes the receptor in an active conformation, facilitating its interaction with coactivators through the LXXLL motifs [6, 7].

Glucocorticoid resistance, also named Chrousos syndrome, is a rare, sporadic or familial condition characterized by biochemically proven hypercortisolism without the clinical stigmata of Cushing syndrome, and by partial or generalized insensitivity to glucocorticoids. Due to this insensitivity, and thereby inadequate negative feedback, serum ACTH, and therefore cortisol production were compensatory stimulated. The chronic excess of ACTH results in an overstimulated steroid biosynthesis, including increased production of adrenal steroids with androgenic and/or mineralocorticoid activity [8, 9]. The clinical spectrum ranges from a completely asymptomatic form [10] to severe, life threatening conditions such as severe hypokalaemia, alkalosis or hypoglycaemia. In addition, hyperandrogenism (acne, hirsutism, infertility, oligo-amenorrhea in females, oligospermia and infertility in males, precocious puberty in children) [11] and mineralocorticoid excess (hypertension and hypokalemic alkalosis) [12] can also be observed. Fatigue is the most common sign of the disease [10]. The diagnosis is based on a detailed evaluation of the hypothalamic-pituitary-adrenal (HPA) axis. Measurement of serum cortisol levels in samples collected in the morning under fasting conditions, at midnight and after dexamethasone administration, together with evaluation of 24 h urinary-free cortisol excretion, are mandatory investigations for diagnosis. Serum cortisol and 24 h urinary free cortisol excretion remain elevated after administration of low dose dexamethasone [13]. Contrary to Cushing’s syndrome, in patients with Chrousos syndrome, the HPA axis preserves its circadian rhythm [13].

To date more than 15 different mutations of the *GR* that cause glucocorticoid resistance have been identified. It has been shown that the mutant receptors may exert a dominant negative effect on the wild-type receptor, or may decrease the receptor’s affinity to the ligand. In addition, a mislocalization of the mutant receptor, delayed or failed translocation to the nucleus or decreased transcriptional activity due to decreased binding through GRE [11] can lead to glucocorticoid resistance.

Here we present the history of a woman evaluated for infertility who carries an already published *GR* gene variant is considered pathogenic. The phenotypes observed in our cases together with those that have been published indicate that the same *GR* gene mutation may present with variable phenotypes, suggesting that other, yet not determined factors, may play a role in development of GR-associated diseases.

## Case presentation

### Patient

A 31-year-old woman presented at our Department due to infertility. Her medical history was unremarkable except unsuccessful attempts for pregnancy for the past 2.5 years. She had regular menstrual cycles since the age of 13 years. On clinical examination, she was normotensive and normokalemic without clinical signs of Cushing’s syndrome or hyperandrogenism. Her height, BMI and glucose homeostasis and bone mineral density proved to be normal (height: 170 cm, BMI: 19.8 kg/m^2^, fasting serum glucose: 5.0 mmol/l and HbA1c: 5.2%), and galactorrhoea was absent. Family history was also unremarkable. Initial laboratory findings indicated an increased serum prolactin level (93 ng/ml; reference range: 1.4–24 ng/ml), but this was due to macroprolactinemia (prolactin recovery after polyethylene glycol: PEG precipitation was 76%). Magnetic resonance imaging did not reveal any pituitary abnormality. A paternal cause of infertility was unlikely because her husband already had two children from his previous marriage. Detailed hormone laboratory investigations of the index patient suggested a partial resistance against glucocorticoids (Table 1). After genetic counseling and written informed consent, Sanger sequencing of the coding region of the *GR* gene (*NR3C1,* NM_000176) was performed. After identification of a pathogenic *GR* mutation, a family screening was indicated for the first degree relatives. Her 35-year-old, clinically healthy sister, who has no fertility problems (mother of a 10-year-old girl) was also genetically tested.

**Table 1:** Laboratory results of the index patient

Parameter	Index patient First visit (range observed during follow-up)	Sister	Reference range
Cortisol (μg/dl)	**32.4 (26–35.4)**	22	8–25
midnight cortisol (μg/dl)	4.8	NA	0–5
morning salivary cortisol (μg/dl)	**1.13 (1.13–1.36)**	NA	< 0.69
midnight salivary cortisol (μg/dl)	0.23 (0.21–0.23)	NA	< 0.43
cortisol after LDDST (μg/dl)	**10.1 (10.1–15.1)**	NA	< 1.8
24 h UFC (nmol/day)	**513** (208–**513**)	NA	100–379
DHEAS (μg/dl)	163 (163–**342**)	204	130–330
ACTH (pg/ml)	**65** (27–**65**)	19.9	7.2–63.3
Androstendione (ng/dl)	**344**	NA	80–280
Prolactin (ng/ml)	**93.5 (25.6–93.5)**	**40.4**	1.39–24.2
Prolactin after PEG recipitation	21 (6.1–21)	NA	1.39–24.2

*LDDST* low dose dexamethasone suppression test, *UFC* urinary free cortisol, *DHEAS* dehydroepiandrosterone sulfate, *ACTH* adrenocorticotrop hormone, *PEG* polyethylene glycol. All cortisol, DHEAS. androstendione measurements were performed from serum, while for ACTH and prolactin plasma was used Abnormal results are highlighted in bold

All patients and family members underwent genetic counseling and informed consent for genetic testing was obtained from all individuals. Evaluation and treatment of human data have been performed in accordance with the Declaration of Helsinki and the study was approved by the Local Ethical Committee of Semmelweis University.

### Laboratory measurements

Laboratory measurements were performed at the Central Laboratory of Semmelweis University. Fasting blood samples were obtained between 08:00 and 09:00 h. Plasma, salivary and urinary cortisol and plasma ACTH, serum estradiol, progesterone, sex hormone binding globulin (SHBG), testosterone, luteinizing hormone (LH), follicle stimulating hormone (FSH), thyroid stimulating hormone (TSH), free thyroxin (fT4), prolactin and growth hormone (GH) concentrations were measured with an electrochemiluminescence immunoassay (Cobas E411, ROCHE, Indianapolis; Architect System, Lisnamuck, Longford, Ireland; IDS-iSYS, Immunodiagnostic Systems Ltd., Boldon, England), while serum dehydroepiandrosterone sulphate (DHEAS) and androstendione concentrations were determined with radioimmunoassay (Beckman Coulter Brea, California, USA).

### Mutation screening of the GR

DNA was isolated from peripheral blood by a standard procedure using commercially available DNA isolation reagents (DNA Isolation kit from blood, Qiagen, San Diego, USA). The whole coding region of the *GR* was evaluated by Sanger sequencing as previously described by Koper et al. [14].

### Hormone laboratory findings

Table 1 summarises the main hormone laboratory findings of the index patient. During repeated measurements, serum cortisol levels in the morning were always elevated (between 26 and 35.4 μg/dl; reference range: 8–25 μg/dl) while plasma ACTH concentration was slightly above the upper limit or within the normal range (between 28.5 and 65 pg/ml; reference range: 7.2–63.3). Morning salivary cortisol levels (determined two times) were also elevated (1.36 and 1,13 μg/dl; reference range: < 0.690) but salivary cortisol collected at midnight was within the reference range (0.21 and 0.23 μg/dl; reference range < 0.430 μg/dl). A low dose (1 mg) overnight dexamethasone suppression test was performed twice, and showed an inadequate suppression of morning serum cortisol (10 and 15 μg/dl; reference range: l < 1.8 μg/dl). Repeated 24 h urinary free cortisol (UFC) concentrations were between 280 and 513 nmol/day (reference range: 100–379). Serum DHEAS was slightly elevated or normal (342 and 163 μg/dl, reference range: 130–330), and serum androstendione was increased (344 ng/dl; reference range 80-280 ng/dl). GH, SHBG, TSH, fT4, LH, FSH, testosterone, progesterone and estradiol levels were all normal (not shown in Table 1).

### Sanger sequencing of the coding region of the GR

**Fig. 1:**
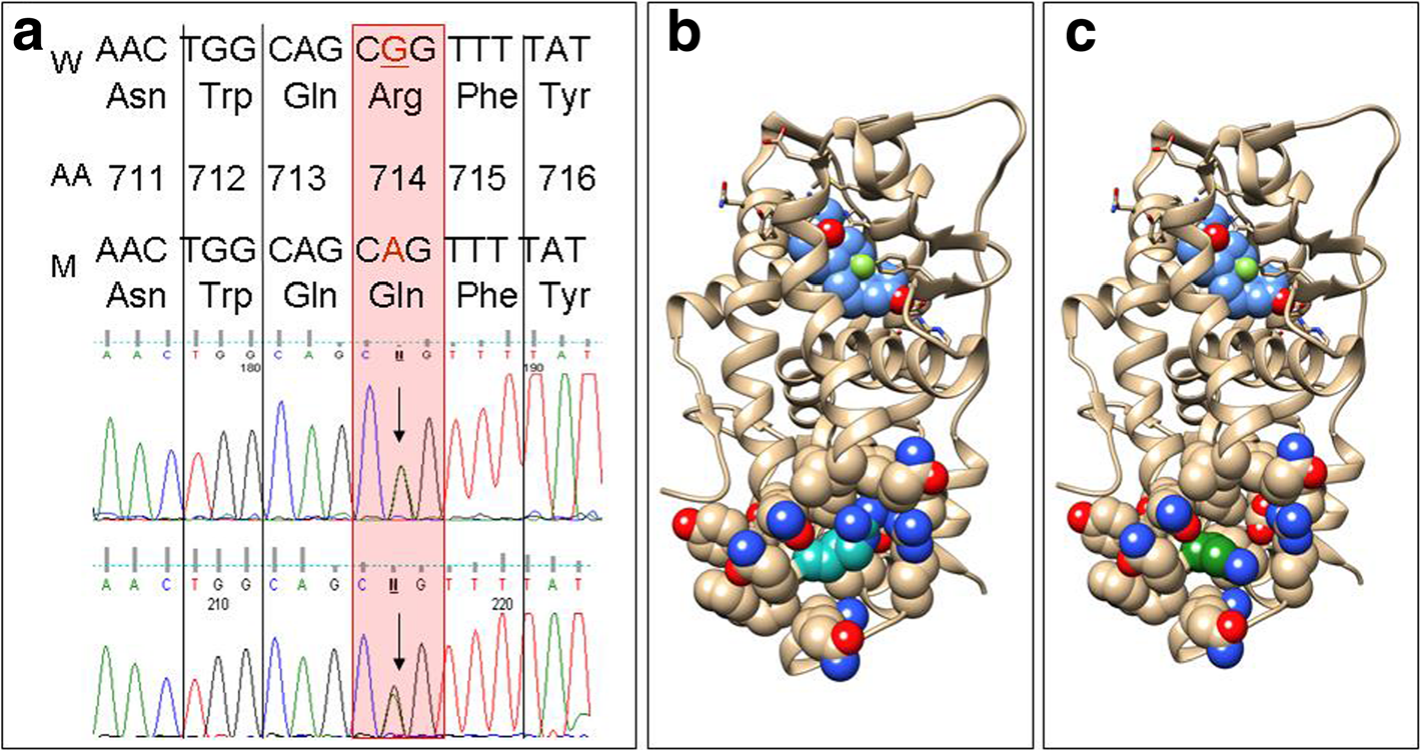
Sanger sequencing and three dimensional model of the Arg and Gln at postion 714 of the GR gene. Chromatogram showing the heterozygous c.2141G➔A mutation of exon 8 of the *GR* gene in the index patient and her clinically unaffected sister (panel **a**); Arginine at position 714 sits on the surface of the domain. The amino acid change from arginine to glutamine may disturb the structure and charge distribution. The GR ligand binding domain coordinates are from PDB structure 4UDC. The domain backbone represented as ribbon. The aminoacid 714 and the surrounding residues are represented with sphere and colored CPK. Arginine side change carbon atoms are colored with light sea green (panel **b**), while glutamine side change carbon atoms are colored with forest green (panel **c**)

As shown in Fig. 1, a heterozygous missense mutation (c.2141G➔A) resulting in a Arg714Gln change was identified in exon 8 of the *GR* gene*.* The same mutation was found in the clinically healthy 35-year-old sister of the patient, who had normal steroid hormone levels. Other family members denied the clinical, genetic or hormonal screening. In addition, this variant was not detected in more than 60 patients and controls tested either for glucocorticoid resistance or Cushing’s syndrome in our Laboratory. Moreover it was not present in commonly used genetic databases including Exome Variant Server (evs.gs.washington.edu/EVS), Exac (exac.broadinstitute.org) and SNPeffect (http://snpeffect.switchlab.org).

### Three-dimensional protein modeling of the Arg714Gln variant of the GR

Molecular modeling and analyses were performed using the UCSF Chimera package [15] (Fig. 1b, c). The coordinates of the GR ligand binding domain have been obtained from PDB structure 4UDC. Arginine at the position 714 is the member of helix 10 of the ligand binding domain (LBD) of the GR. It locates opposite side of the ligand binding pocket and relatively far from any known functional region. However, arginine has a large, positively charged side chain, which protrudes into a space created by helices 7–10 (Fig. 1), but glutamine has a smaller, uncharged side chain, which may release helix 10 from its original position, which may lead to further conformational changes in the ligand-binding pocket. Nader et al. performed a complex functional testing of this mutation and using the quantification of the thickness of both the wild type and mutant Cα showed that the mutant LBD had an increased distance in root mean square deviation over the duration of the simulation compared to the wild type receptor, suggesting that the mutant structure binds the peptide with less affinity [16].

## Discussion and conclusions

In the present study, we identified a missense (R714Q) variant of the *GR*gene in a heterozygous form in a young woman evaluated for infertility. It is particularly interesting that the initial hormonal finding in our patient revealed hyperprolactinemia, which is a well-known cause of infertility. However, hyperprolactinaemia was excluded as a cause of infertility based on the results of PEG-precipitation, the lack of clinical signs and magnetic resonance imaging.

During detailed endocrinological investigation for the cause of infertility, we demonstrated that our patient has a mild resistance against glucocorticoids. Sanger sequencing of the coding region of the gene encoding *GR* was performed and an already described variant was identified. The pathogenetic role of the R714Q variant of the *GR* gene has already been suggested by Nader et al. who found this variant in a 2-year-old girl presenting with hypoglycaemia, hypokalaemia, hypertension, premature pubarche, mild clitoromegaly, advanced bone age, elevated cortisol, ACTH, DHEA, androstenedione and urinary 17-ketosteroid levels [16]. In addition, the functional consequences of the mutant receptor was also confirmed, as the mutant receptor displayed a decreased transcriptional activity with a 2-fold reduction in affinity to ligand, and a dominant negative effect on the wild-type receptor [16]. Molecular modeling demonstrated that substitution of arginine by glutamine in the 714 position of the glucocorticoid receptor may cause conformational changes of the ligand-binding pocket, and the AF-2 domain, leading to an approximately 2-fold reduction in affinity to ligand [7].

The phenotype difference observed between the published case and ours highlights and again confirms that the clinical manifestation of glucocorticoid resistance is very heterogeneous (Table 2), and the same mutation may lead to both severe and mild, even clinically insignificant manifestations.

**Table 2:** Clinical signs of glucocorticoid resistance in previously reported cases

Author [reference]	Age (years)	Sex	GR mutation	Clinical signs
Chrousos et al. 1982 [8]	58	Male	c.2054A > T, D641V	hypertension, hypokalemia
Brönnegard et al. 1986 [17]	46	Female	NA	fatigue
Karl et al. 1993 [18]	26	Female	4 bp deletion in exon 6	hirsutism, Male-pattern baldness, menstrual irregularities
Malchoff et al. 1993 [19]	6–7	Male	c.2317G > A, V729I	precocious puberty, hyperandrogenism
Karl et al. 1996 [20] and	33	Male	c.1808 T > A, I559N	infertility
Kino et al. 2001 [21]	38	Male	c.1808 T > A, I559N	ACTH producing pituitary adenoma in the same patient
Ruiz et al. 2001 [10]	41	Female	c.1430G > A, R477H	hirsutism, fatigue, obesity
Ruiz et al. 2001 [10]	31	Female	c.2035G > A, G679S	hirsutism
Mendonca et al. 2002 [22]	1 day	Female	homozygous c.1844 T > C, V571A	Female pseudohermaphroditism, ambiguous genitalia, hypertension, hypokalaemia
Vottero et al. 2002 [23]	18	Female	c.2373 T > G, I747M	cystic acne, hyperandrogenism, hirsutism, oligomenorrhoea
Charmandari et al. 2005 [24]	29	Female	c.2318 T > C, L773P	fatigue, anxiety, acne, hirsutism
Charmandari et al. 2007 [25]	7	Male	c.2209 T > C, F737 L	hypertension, hypokalaemia
Charmandari et al. 2008 [11]	43	Female	c.1201G > C, D401H	tissue-specific glucocorticoid *hypersensitivity,* obesity, hypertension, type 2 diabetes, metabolic syndrome
Raef et al. 2008 [26]	19	Male	c.2035G > A, G679S	hypertension, hypokalaemia, precocious puberty
Nader et al. 2010 [15]	2	Female	c.2141G > A, R714Q	hypoglycaemia, hypokalaemia, hypertension, premature pubarche, mild clitoromegaly
McMahon et al. 2010 [27]	1 day	Male	c.2318_2319delTG, Leu773fs	Hypoglycaemia, fatigue, hypertension
Trebble et al. 2010 [28]	20	Female	c.1835delC, Arg612fs	fatigue, facial hirsutism

Treatment of glucocorticoid resistance includes administration of a high dose of glucorcorticoids in order to suppress the excessive ACTH-stimulated secretion of mineralocorticoids and androgens [13]. However, our patient had no clinical symptoms of mineralocorticoid or androgen excess, and it is not known whether a high dose of glucocorticoids could offer an option for the treatment of infertility with an acceptable maternal and fetal risk, and whether glucocorticoids should be continued during pregnancy. Because the Arg714Glu variant of the *GR* gene may cause both mild and severe phenotypes, a high dose of glucocorticoids may be of value to prevent fetal androgen and mineralocorticoid excess in an affected fetus predisposed to a severe phenotype. Detailed genetic counseling is indicated and prenatal genetic testing possible in order to determine the fetus genotype.

In summary, we present a 31-year-old woman who was evaluated for infertility and was diagnosed with a mild phenotype of glucocorticoid resistance caused by the previously identified, pathogenic Arg714Gln mutation of the *GR* gene. Its pathogenicity has been suggested by Nader et al. who identified this variant in a child with a severe disease phenotype [16]. However, we found this variant in the clinically healthy sister of our index case, suggesting that the Arg714Gln mutation, may lead to mild diseases or it can be clinically insignificant too. Other mechanisms or modifier genes may explain the phenotype heterogeneity of *GR*-associated phenotypes.

## Abbreviations

Arg714Glu: arginine➔glutamine change at the 714 nucleic position
c2141G➔A: guanine➔adenine change at the 2141 position
R714Q: arginine➔glutamine change at the 714 nucleic position
ACTH: Adrenocorticotropic hormone
AF-1: Activation function 1
AF-2: Activation function 2
BMI: Body mass index
DBD: DNA binding domain
DHEA: Dehydroepiandrosterone
DHEAS: Dehydroepiandrosterone sulfate
DNA: Deoxyribonucleic acid
FSH: Follicle stimulating hormone
fT4: Free thyroxin
GH: Growth hormone
GR: Glucocorticoid receptor
GRE: Glucocorticoid response element
GRIP1: Glucocorticoid receptor interacting protein 1
HPA: Hypothalamic-pituitary-adrenal axis
HSP90: Heat shock protein 90
LBD: Ligand binding domain
LH: Luteinizing hormone
NTD: N-terminal transactivation domain
p160: protein160
PCR: Polymerase chain reaction
PEG: Polyethylene glycol
RNA: Ribonucleic acid
SHBG: Sex hormone binding globulin
TSH: Thyroid stimulating hormone
UFC: Urinary free cortisol
